# Mechanically activated switching of Si-based single-molecule junction as imaged with three-dimensional dynamic probe

**DOI:** 10.1038/ncomms9465

**Published:** 2015-10-06

**Authors:** Miki Nakamura, Shoji Yoshida, Tomoki Katayama, Atsushi Taninaka, Yutaka Mera, Susumu Okada, Osamu Takeuchi, Hidemi Shigekawa

**Affiliations:** 1 Faculty of Pure and Applied Sciences, University of Tsukuba, Tsukuba 305-8571, Japan; 2 Shiga University of Medical Science, Shiga 520-2122, Japan

## Abstract

Understanding and extracting the full functions of single-molecule characteristics are key factors in the development of future device technologies, as well as in basic research on molecular electronics. Here we report a new methodology for realizing a three-dimensional (3D) dynamic probe of single-molecule conductance, which enables the elaborate 3D analysis of the conformational effect on molecular electronics, by the formation of a Si/single molecule/Si structure using scanning tunnelling microscopy (STM). The formation of robust covalent bonds between a molecule and Si electrodes, together with STM-related techniques, enables the stable and repeated control of the conformational modulation of the molecule. By 3D imaging of the conformational effect on a 1,4-diethynylbenzene molecule, a binary change in conductance with hysteresis is observed for the first time, which is considered to originate from a mechanically activated conformational change.

## Abstract (web summary)

Mechanically induced conformational modulation can be used to control the conductance of single molecules junctions, but it is hard to be realized due to broken junctions. Here, the authors probe three-dimensional dynamics of Si/single-molecule/Si junctions, whose conductance shows a binary change.

## Graphical abstract

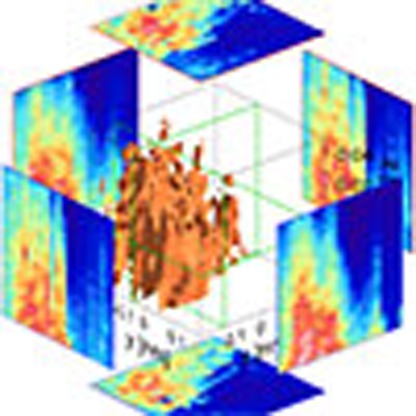

The understanding of a variety of single-molecule characteristics, such as rectification, negative resistance, switching, spin properties and flexibility, has been a major concept in developing future device technologies, as well as in basic research on molecular electronics^1^,^2^,^3^,^4^,^5^,^6^,^7^,^8^,^9^,^10^,^11^,^12^,^13^,^14^,^15^,^16^,^17^,^18^,^19^,^20^,^21^,^22^,^23^. As pioneering works, measurements, such as by the break junction method, of metal/single molecule/metal structures have revealed various intriguing molecular characteristics^9^,^10^,^11^,^12^,^13^,^14^,^15^,^16^,^17^,^18^,^19^,^20^,^21^,^22^,^23^. In this decade, the importance of mechanically activated processes has been recognized and the study of their effects has been attracting considerable attention^13^,^14^,^15^,^16^,^17^,^18^. A new type of mechanism has been proposed theoretically to explain molecular conductance switching, in which mechanically activated dynamic bistability in molecular conformations and its effect on molecular electronic structures play an essential role^17^. Towards the understanding and extraction of the full functions of molecules, dynamic analysis of the conformational effect is a key factor.

Here we report a new methodology for realizing a three-dimensional (3D) dynamic probe of single-molecule conductance, which enables elaborate 3D analysis, by the formation of a Si/single molecule/Si structure using scanning tunnelling microscopy (STM). The formation of robust covalent bonds between a molecule and Si electrodes^24^,^25^, together with STM-related techniques, enables the stable and repeated control of the conformational modulation of the molecule. A binary change in conductance with hysteresis, which is considered to originate from a mechanically activated conformational change, is observed for the first time.

## Results

### 3D dynamic probe STM

**Figure 1:**
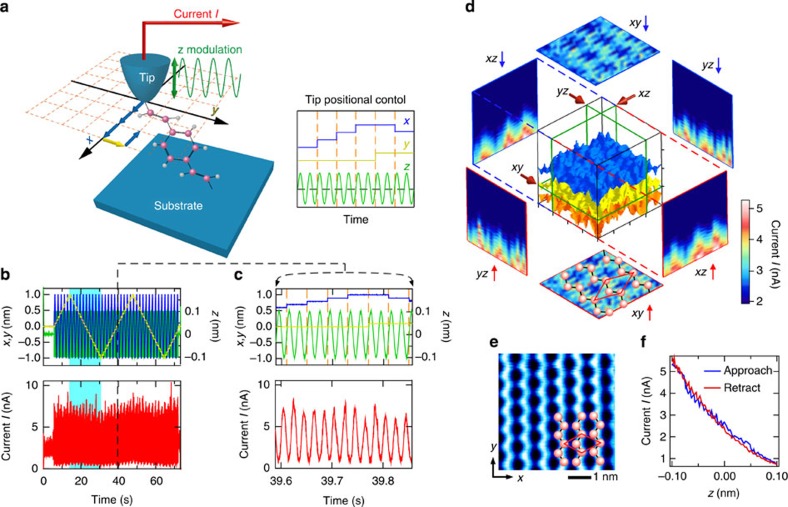
3D measurement system and results for Si(001) surface. (**a**) Schematics of measurement setup (left) and tip control scheme. The STM tip, which was moved back and forth in the *z* direction in accordance with a sinusoidal function (green), was scanned two-dimensionally in the *x* (blue) and *y* (yellow) directions, as shown in the scheme. (**b**,**c**) Change in current (red) obtained for a Si(001)-c(4 × 2) surface and its magnification. Measurement was carried out over a scanning range with Δ*x*, Δ*y* and Δ*z*=2, 2 and 0.2 nm, respectively, at 21 × 21 × 200 mesh points. (**d**) 3D map of acquired current. The equicurrent surfaces of *I*_t_=1.5, 2.5 and 3.5 nA are shown in the central cube. The cross-sectional *xy*, *yz* and *zx* planes show 2D maps of the current obtained at the 21 × 21 (*xy* plane) and 21 × 200 (*yz* and *zx* planes) mesh points on the plane indicated by the arrows and the green frames drawn on the cube, where, for example, *xy*↑ and *xy*↓indicate that the data in cross sections were obtained while the STM tip was retracted from and made to approach the Si surface, respectively. (**e**) STM image and schematic of a surface with a c(4 × 2) phase, whose structural model is also drawn in the *xy*↑ plane in **d**. (**f**) Tunnelling current as a function of the STM tip-substrate distance. The cross sections of *xz*↑ and *yz*↑ are similar to those of *xz*↓ and *yz*↓, respectively.

The 3D measurement system is schematically shown in Fig. 1a. The current *I* flowing through a single-molecule junction with a Si STM tip/single molecule/Si substrate structure was measured with a fixed bias voltage *V*_s_ applied between the STM tip and the substrate, while the STM tip, which was moved back and forth in the *z* direction in accordance with a sinusoidal function, was scanned two-dimensionally (*x* and *y* directions) (see Methods section). Figure 1b–d show an example of data that were acquired over a bare Si(001) surface at 77 K without a molecule between the STM tip and the substrate to examine our newly developed system. The blue, yellow, green and red lines in Fig. 1b indicate the *x* and *y* scans, *z* modulation and current, respectively. Figure 1c shows a magnification of Fig. 1b. The data in the light blue region in Fig. 1b (21 × 21 × 200 measurement points in the *x*, *y* and *z* directions) were analysed and are shown in Fig. 1d. Here the equicurrent surfaces of *I*_t_=1.5, 2.5 and 3.5 nA are shown in the central cube. The cross-sectional *xy*, *yz* and *zx* planes obtained at the positions indicated by the red arrows and green frames drawn on the cube are shown together, in which arrows such as *xy*↑ and *xy*↓indicate that the data in the cross sections were obtained while the tip was retracted from and made to approach the Si surface, respectively. For the cross sections, all the data in each plane were used to produce each map with the color scale. Figure 1e shows an STM image obtained for a Si surface with a c (4 × 2) phase; its structural model is also drawn in the *xy*↑ plane in Fig. 1d (ref. 26). As expected, the three equicurrent surfaces in the cube in Fig. 1d are parallel to the Si surface except for the c(4 × 2) atomic structure, and simple exponential decay of the tunnelling current was observed with increasing tip-sample distance along the *z* direction (Fig. 1f).

### Sample preparations

**Figure 2:**
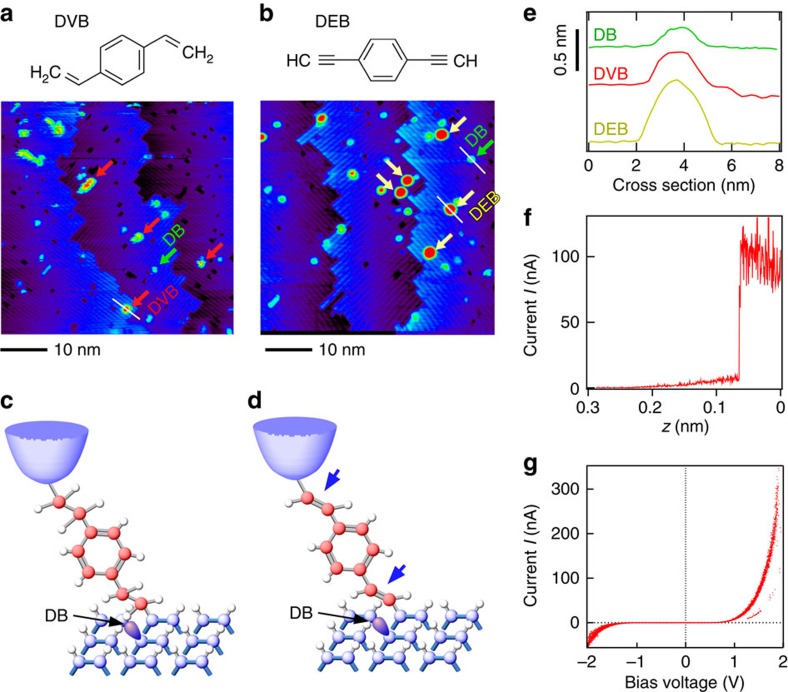
Si/single molecule/Si junction. (**a**,**b**) STM images of H-terminated Si(100) (H-Si(100)) surface after dosing small amount of DVB and DEB molecules at room temperature, respectively. (**c**,**d**) A Si/DVB (DEB) molecule/Si junction was formed via the approach of a Si STM tip toward an isolated DVB/DEB molecule after confirming the sufficient isolation by STM. The double (triple) bond at the free end of the DVB (DEB) molecule covalently reacts with Si-dangling bond (Si-DB) on the STM tip. (**e**). Cross sections obtained for DB, and for DVB and DEB molecules. (**f**). Current as a function of the STM tip-substrate distance, where the jump in current indicates the formation of a bond. (**g**) Twenty *I–V* curves repeatedly measured for a DEB molecule. Here all measurements were carried out at room temperature.

After the system was examined using a bare Si surface, we carried out similar 3D measurements to study the effect of different molecular conformations on single-molecule conductance. We realized a robust single-molecule junction by forming rigid Si–C bonds between a molecule and electrodes consisting of a Si STM tip and a Si substrate (see Methods section)^27^,^28^,^29^. 1,4-divinylbenzene (DVB) and 1,4-diethynylbenzene (DEB) schematically shown in Fig. 2a,b, respectively, were chosen as the single molecules to examine the conformational effect on the electronic structures. The double and triple bonds at both ends of the DVB and DEB molecules, respectively, make them suitable for the study of Si/single molecule/Si junctions and for the comparison of results. Isolated DVB or DEB molecules were prepared on a H-terminated Si(001) surface. On the basis of the reaction reported in previous papers for styrene and phenylacetylene molecules on H–Si(001)^30^,^31^, an adsorbed DVB (DEB) molecule is considered to be tilted from the direction normal to the Si(001) substrate surface, as shown in Fig. 2c,d. The difference in the molecular height in the STM image observed when using DVB and DEB shown in Fig. 2e indicates a difference in the conductance of these molecules depending on the existence or nonexistence of double bonds in the junction^31^. A Si STM tip/single molecule/Si substrate junction was formed by the point contact method^11^ (Methods section). Stable contact between the STM tip and the DVB or DEB molecule was confirmed by the abrupt change in current (Fig. 2f) and the repeatability of *I*–*V* curve measurement (Fig. 2g). Although these measurements were carried out at room temperature, similar *I–V* curves were stably reproduced in measurements, showing the reliability of the following analysis. Hereafter, measurements were carried out at 78 K (see Methods section).

### 3D imaging of molecular dynamics

**Figure 3:**
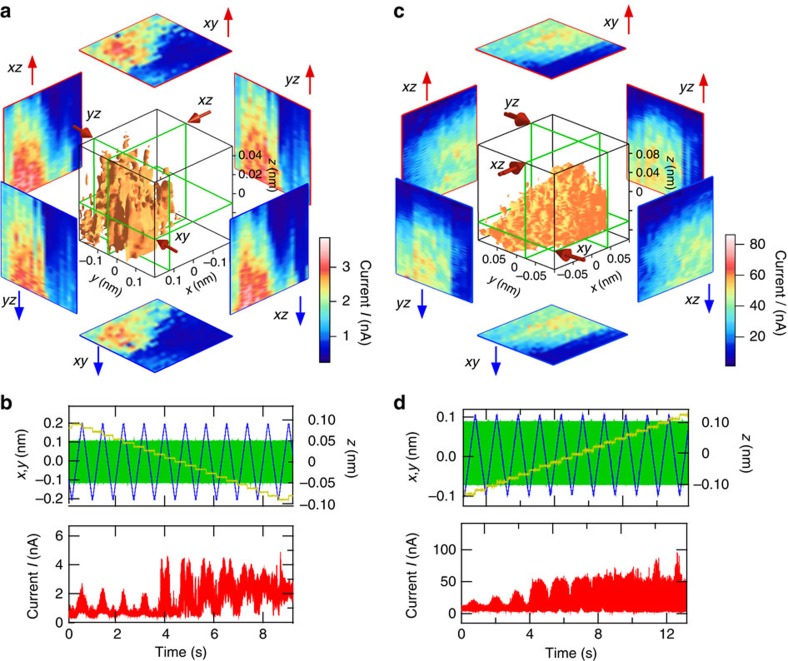
3D dynamic measurement using Si/DVB(DEB)/Si single-molecule junctions. (**a**,**b**) and (**c**,**d**) are 3D maps and their raw data obtained for DVB and DEB molecules, respectively. Here the equicurrent surfaces of *I*_t_=2.5 and 40 nA are drawn in the cubes corresponding to DVB and DEB molecules, respectively.

Figure 3a,c show 3D maps of the data shown in Fig. 3b,d, respectively. Here the equicurrent surfaces of *I*_t_=2.5 and 40 nA are drawn in the cubes corresponding to the DVB (Fig. 3a) and DEB (Fig. 3c) molecules, respectively. The equicurrent surfaces are almost smooth and have a roughly hemispherical contour, which is different from the 3D map obtained for the bare Si surface without a molecule shown in Fig. 1d. As can be seen in the cross sections, the change in current owing to *z* modulation is smooth for both molecules over the scanning range, indicating a reasonably smooth change in the conductance caused by the structural change in this range. The smooth change in current obtained at neighbouring measurement points over the surface indicates the high reproducibility of this process.

The intensity of the current observed for the DEB molecule was one order larger than that for the DVB molecule, as shown in Fig. 3b,d, which is consistent with the above mentioned fact that a double bond remained at both ends of the junction in the case of the DEB molecule, producing a higher conductance. Experiments carried out for 220 DVB molecules showed the smooth change in the current shown in Fig. 3b,d in this scan range. In contrast, another type of change in conductance was observed in 20% of the measurements using 103 DEB molecules.

### Binary change in conductance with hysteresis

**Figure 4:**
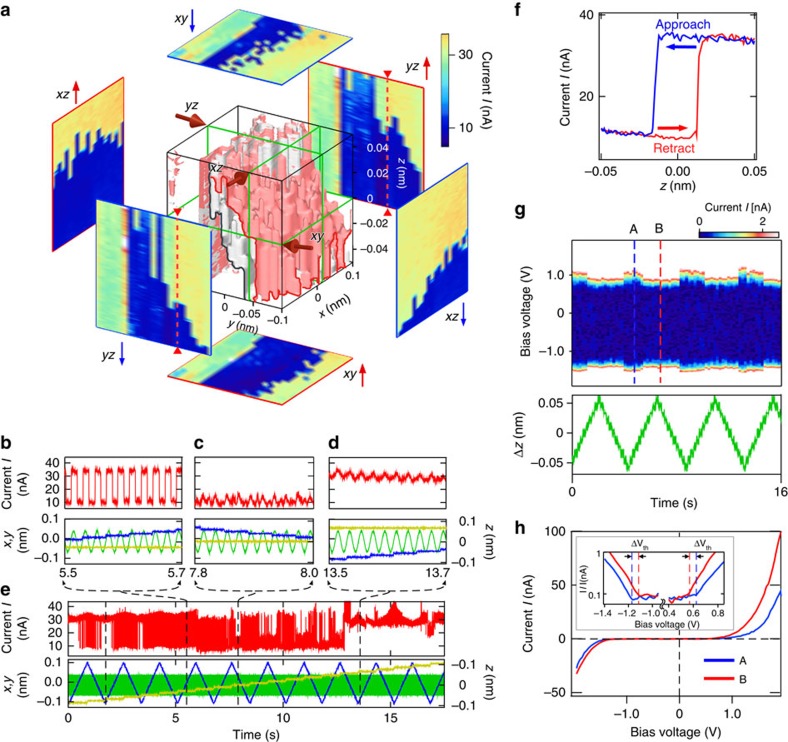
Hysteresis characteristics observed for Si/DEB/Si single-molecule junction. (**a**) 3D map, where the equicurrent surfaces of *I*=20 nA for the STM tip being retracted (red) and approached (grey) are shown in the cube. (**b**–**e**) Raw data (**e**) and magnifications of three parts of **e** (**b**–**d**). (**c**,**d**) Show the two different states with high and low currents, respectively. (**b**) shows the change between the two states caused by *z* modulation. (**f**) *I–z* curves measured for the STM tip being retracted and approached, showing hysteresis, which corresponds to the change at the position marked by the red small triangles on the *xy*↑ and *xy*↓ cross sections. (**g**) Color scale image of *I–V* curves (upper), taken at each step in the *z* direction (green lines in the lower graph) (see Methods section). (**h**) *I–V* curves obtained at A and B in **g**. The inset is current in a logarithmic form, ln|*I*|, which shows the rising point of current. Δ*V*_th_ indicates the shift of the rising point of *I–V* curve.

Figure 4 shows an example of the other type of change observed for the DEB molecules. Figure 4a shows a 3D image of the data in Fig. 4e. Figure 4b–d show magnifications of the three regions of Fig. 4e. As shown in Fig. 4c,d, there are two different states producing two different currents. In addition to the smooth change in current while remaining in the two states, an abrupt change in current between the two states was observed during *z* modulation, as shown in Fig. 4b, which can be clearly seen as the abrupt change between the blue and yellow colours in the *xz* and *yz* cross sections in Fig. 4a.

Furthermore, the difference in the positions where the abrupt change occurs, for example, between the *yz*↑ and *yz*↓ cross sections, indicates the existence of hysteresis in this process. Namely, the abrupt change occurs at different *z* positions when the STM tip is being retracted from and made to approach the substrate. Figure 4f shows an example obtained at the position marked by the red small triangles on the *yz*↑ and *yz*↓ cross sections. In Fig. 4a, the equicurrent surfaces of *I*=20 nA for the STM tip being retracted (red) and approached (grey) are shown in the cube. The distance between the two equicurrent surfaces shows the characteristic of hysteresis over the surface.

To clarify the origin of the observed change in current, we obtained *I–V* curves as a function of *z* modulation (see Methods section). Figure 4g shows a color scale graph of a series of *I–V* curves, which shows the change in the initial point where the *I–V* curves start to rise. A change in the *I–V* curves that depended on the electrode-gap distance, that is, the existence of the two states, is clearly observed. Figure 4h shows typical *I–V* curves obtained at two different electrode-gap distances (A and B in Fig. 4g), where the rising point is different for the two states, as shown in the logarithmic plot in the inset. On the basis of the results of the theoretical analysis of the junction with semiconductor electrodes, in which the existence of a band gap affects the conductance characteristics differently from the cases of metal electrodes^32^,^33^, the difference in the rising point of the *I–V* curve is considered to reflect the difference in the position of lowest unoccupied molecular orbital (LUMO)/highest occupied molecular orbital energy level^29^,^34^ and the dominant current is caused by the LUMO level in the case of the DEB molecule^27^. The observed phenomena are consistent with the results of analysis. The slight asymmetry in the *I–V* curves is considered to be produced by the STM tip and the substrate in the junction.

**Figure 5:**
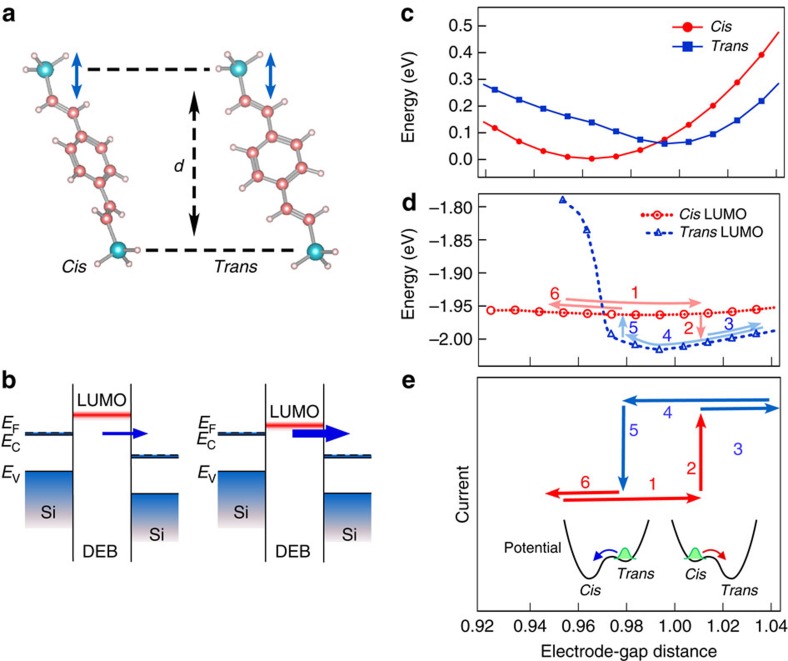
Results of theoretical analysis on the molecular electronic structures associated with the modulation of electrode-gap distance. (**a**) *Cis* and *trans* conformations of DEB molecule formed in the Si/DEB molecule/Si junction. The electrode-gap distance *d* in the direction perpendicular to the substrate (indicated by arrows) was changed in steps of 0.01 nm. (**b**) Schematic illustrations of band structures used to explain the change in tunnelling current. (**c**) Structural energies of *cis* and *trans* conformations calculated using Gaussian 03. (**d**) Change in LUMO levels for *cis* and *trans* conformations as a function of electrode-gap distance. (**e**) Schematic showing the change in current (the hysteresis in Fig. 4), where the numbers correspond to those in **d**. A potential model for the change is also shown.

The observed binary switching of conductance between the two current states is based on the robust Si–C covalent bonds and has a reproducible hysteresis characteristic. Furthermore, once a change from a low- to high-current state or from a high- to low-current state has occurred during a change in the electrode-gap distance, the reverse change does not occur without the opposite change in the electrode-gap distance, even when the bias voltage is changed between −2.0 and +2.0 V, which suggests that the change is not simply induced by the bias voltage or tunnelling current^35^,^36^,^37^,^38^. Namely, the mechanism underlying the observed characteristics is not simply similar to those proposed in previous works, such as the continuous modulation of *π* orbitals and the change in adsorption sites on electrodes^11^,^14^,^39^. The DEB molecule originally has a symmetric structure similarly to the DVB molecule, as shown in Fig. 2b. However, the DEB molecule becomes asymmetric on bonding with Si electrodes, that is, it can have *cis* and *trans* conformations after losing the triple bonds at both its ends, as shown in Fig. 5a, which may produce the observed characteristics.

### Theoretical analysis of the binary change

To examine the process in more detail, theoretical calculations were carried out (see Methods section). Here a one-dimensional change was examined. The total energy of the DEB molecule as a function of electrode-gap distance (*d* in Fig. 5a) is shown in Fig. 5c. The minimum energies of the *cis* and *trans* conformations are at electrode-gap distances of ∼0.96 nm and ∼1.0 nm, respectively, and the two curves intersect, indicating the possibility that strain induces a conformational transformation from *cis* to *trans* and vice versa depending on the electrode-gap distance.

Since n-type Si is used for the STM tip and substrate, when the LUMO level approaches the conduction band edge of Si, a higher current is expected via the LUMO level^27^, as shown schematically in Fig. 5b. Figure 5d shows the shift in the LUMO levels of the two conformations as a function of electrode-gap distance. The LUMO level of the *trans* conformation for long electrode-gap distances is lower than that of the *cis* conformation. Namely, the LUMO level has a low-current state for the *cis* conformation and a high-current state for the *trans* conformation. The difference in the energy level of LUMO in Fig. 5d at the electrode-gap distance corresponding to the two minimum energy points in Fig. 5c (−1.962 eV at ∼0.96 nm and −2.017 eV at ∼1.0 nm) is ∼55 meV, which is consistent with the shift of the rising point of the *IV* curve (Δ*V*_th_) shown in Fig. 4h, ∼78 meV.

## Discussion

A possible structural change that may produce the observed hysteresis loop and the associated change in current is shown in Fig. 5e. When the electrode-gap distance is increased from arrow 1 to arrow 3, the conformation changes from *cis* to *trans* at the point indicated by arrow 2, and then back from *trans* to *cis* at the point indicated by arrow 5 with decreasing electrode-gap distance (arrow 4 to arrow 6). Accordingly, the current abruptly increases with the structural change from the *cis* conformation to the *trans* conformation (arrow 2), and abruptly decreases when the structure changes from the *trans* conformation to the *cis* conformation (arrow 5). This change in the electronic structure is consistent with the experimental result, that is, the change in conductance with hysteresis shown in Fig. 4. The variation in the switching point is considered to be due to the stochastic process of the conformational change with a potential energy barrier shown in Fig. 5e.

The observed positional (*x* and *y*) and molecular dependences of the hysteresis characteristics may be due to the initial conformation of the junctions. For example, the molecules deposited on a Si substrate have a degree of rotational freedom along the axis of the Si–C bond before the formation of a bond with the STM tip apex, and although we verified the atomic resolution before forming the junction, that is, the quality of the STM tip, the atomic structure of the Si STM tip apex cannot be well defined at this stage. To enable further analysis, more detailed calculations for the 3D conformational change are necessary, which are left as future work.

In conclusion, a new methodology of realizing the 3D dynamic probe of single-molecule conductance, which enables elaborate analysis, was demonstrated. A binary change in conductance with hysteresis, which is considered to originate from a mechanically activated conformational change, was observed for a DEB molecule. As a possible mechanism, a *cis*–*trans* conformational change was discussed. The formation of stable covalent bonds between a molecule and semiconductor electrodes, together with the use of high-controllability semiconductor technologies, is expected to enable both basic research and the fabrication of a variety of molecular devices^24^,^25^,^28^,^29^,^30^,^31^,^40^,^41^. By choosing suitable molecules, new functions originating from the flexibility of molecules, which solid-state devices themselves may not have, have the potential to be actively used for the formation of integrated functions.

## Methods

### Sample and tip preparation

A STM tip and a substrate were prepared from the same n-type Si(001) wafer (As-doped, 0.003±0.001 Ωcm). The STM tip cut out of the wafer (∼1 × 3 × 0.3 mm^3^), one end of which was cut along a diagonal line, was attached to a Mo holder and cleaned by heating with an electron beam at ∼1,100 °C. The Si substrate was cleaned by resistive heating and the quality of the surface was confirmed by taking STM images, as shown in Fig. 1e. After confirming the quality of the hydrogen-terminated Si surface by STM, DVB or DEB molecules (Sigma-Aldrich, 86 and 96% purity for DVB and DEB, respectively) were deposited on the surface at room temperature. For all measurements, a STM tip that provides a STM image with atomic resolution, as shown in Figs 1e and 2a,b, was used.

### Measurements

We have improved the point contact method of STM^11^ to enable 3D dynamic analysis of the effect of different molecular conformations on the conductance in a single-molecule junction. In each measurement, we moved the STM tip towards an isolated molecule from just above it after observing the target molecule at atomic resolution. The observed current abruptly increases when a junction is formed. The probability for the formation of a junction was as low as 10% in this experiment, probably owing to the relative positional relationship between the bonds of Si and the molecule, which also reduces the probability of the formation of a multimolecule junction. After confirming the chemical contact between the STM tip and a DVB or DEB molecule, a 3D dynamic measurement of conductance was carried out using an Omicron LT-STM at 78 K. Samples were kept at a 78 K for 2–3 days before measurements to reduce thermal drift. Then, the current *I* (*x*, *y*, *z*, *V*_s_) measured at each (*x*, *y*, *z*) point (21 × 21 × 200 mesh points) with a bias voltage of *V*_s_ was three-dimensionally mapped using a color scale depending on the purpose, to illustrate the relationship between the 3D molecular conformation and its conductance.

To obtain the *I–V* curves shown in Fig. 4g, the STM tip was reversibly moved ±0.05 nm up and down from the contact point using a step-like function (0.011 nm per step) instead of the sinusoidal function, as shown by the green line in Fig. 4g. I-V measurement was carried out after each step-like change in the electrode-gap distance, where the bias voltage was changed between −2.0 and +2.0 V in each measurement.

### Calculation of electronic structures

The electronic structures of the DEB molecule in the *cis* and *trans* conformations were calculated using Gaussian 03 in the DFT method with the B3LYP hybrid functional, which is most commonly used for the analysis of molecular systems^42^,^43^,^44^, in which the HF functional, Slater functional and Becke functional based on the generalized gradient approximation^43^ are used as exchange functionals, and the Lee–Yang–Parr functional and Vosko–Wilk–Nusair functional are used as correlation functionals. Correlation-consistent polarization plus valence double-zeta basis sets, which are widely used for the calculation of molecules adsorbed on Si surfaces^44^, were used for H, C and Si atoms.

Both ends of the *cis* and *trans* conformations of the DEB molecule were terminated by SiH_3_. The molecule was placed at an angle that reproduces the conformation shown in Fig. 2, and the electrode-gap distance in the direction perpendicular to the substrate (indicated by arrows in Fig. 5a) was changed in steps of 0.01 nm. The bond length between Si and H was fixed at the 0.148 nm observed in SiH_4_, and the geometry of the molecule was optimized for each step-like change in the electrode-gap distance. The structural energy and the position of the LUMO level were calculated for each optimized structure.

## Additional information

**How to cite this article:** Nakamura, M. *et al*. Mechanically activated switching of Si-based single-molecule junction as imaged with three-dimensional dynamic probe. *Nat. Commun.* 6:8465 doi: 10.1038/ncomms9465 (2015).
